# Enhanced stabilization of cellulose-lignin hybrid filaments for carbon fiber production

**DOI:** 10.1007/s10570-017-1579-0

**Published:** 2017-12-01

**Authors:** Nolene Byrne, Rasike De Silva, Yibo Ma, Herbert Sixta, Michael Hummel

**Affiliations:** 1 Institute for Frontier Materials, Deakin University, Geelong, VIC 3216 Australia; 2 Department of Bioproducts and Biosystems, School of Chemical Engineering, Aalto University, PO Box 16300, 00076 Aalto, Finland

**Keywords:** Cellulose-lignin hybrid fibre, Carbon fibre, Stabilization

## Abstract

Herein we investigate the stabilization behavior of a cellulose-lignin composite fibre towards application as a new bio derived precursor for carbon fibres. Carbon fibre materials are in high demand as we move towards a lower emission high-efficiency society. However, the most prominent current carbon fibre precursor is an expensive fossil-based polymer. Over the past decade significant research has focused on using renewable and bio derived alternatives. By blending cellulose and lignin and spinning a fibre with a continuous bi-component matrix a new approach to overcome the current limitations of both these precursors is proposed. A thorough study is conducted here on understanding the stabilization of the new precursors which is a critical step in the carbon fibre process. We show that stabilization times of the composite fibre are significantly reduced in comparison to pure lignin and improvements in mass yield compared to pure cellulose fibres are observed.

## Introduction

Carbon fibre (CF) composites are increasingly important for applications which require lightweight solutions (Frank et al. 2014). CFs are typically embedded in an epoxy or other polymer matrix to create CF-reinforced composite materials (Bekyarova et al. 2007; Meier 1995; Morgan 2005). CF composites are extensively used in aeronautics, aerospace, high-end sports equipment, and racing/sport and luxury cars (Glowacz 2015; Morgan 2005). The demand for CF composites is high, however the prohibitive costs have so far limited their use in mainstream automotive applications, construction and energy sector where their implantation would result in significant improvements regarding energy efficiency and reductions in CO_2_ emissions. The cost of CF composites is largely based on the high price of CFs. They are currently produced predominately from polyacrylonitrile (PAN) with small amounts made from pitches, notably mesophase (Baker and Rials 2013). PAN is an expensive petroleum derived polymer which is typically processed via solution spinning into high quality filaments. Subsequent CF manufacture requires this starting fibre (precursor) to be gently heated through a series of ovens and furnaces which is a time and energy intensive process and contributes considerably to the total cost of the CF (Buckley and Edie 1993; Fitzer 1989; Windhorst and Blount 1997). Current research efforts are thus divided between finding alternative precursor materials and developing more efficient methods for their conversion into CF (Byrne et al. 2014b; Chand 2000; Frank et al. 2014).

Renewable polymers like cellulose and lignin are both regarded as excellent candidates for CF largely due to the lower cost of the starting material (Baker and Rials 2013; Dumanlı and Windle 2012; Kadla et al. 2002; Sadeghifar et al. 2016). Indeed both cellulose and lignin based CF have been commercially produced at one point in the past (Bacon and Tang 1964; Dumanlı and Windle 2012). The major limitation preventing largescale production of either cellulose or lignin differs. Upon pyrolysis of cellulose, the maximum carbon yield of 44.4% is rarely reached. Often, substantially lower yields are observed unless the precursor fibre is impregnated with catalysts promoting the dehydration reaction (Byrne et al. 2014a; Kandola et al. 1996; Zhou et al. 2016). In combination with the limited mechanical properties that were offered by the first generation of (viscose-type) continuous cellulose filaments, cellulose-derived CFs seemed little promising and were replaced rapidly by PAN in the 1960s (Jenkins and Kawamura 1976; Savage 2012). Lignin, on the other hand, has a mass yield of 55% comparable with PAN but due to its thermoplastic nature often requires extensive stabilization times, up to 100 h (Hosseinaei et al. 2016; Oroumei et al. 2015). This long stabilization time and challenges in the melt spinning of lignin without plasticising measures, and still limited mechanical properties of the resulting CF prevented large scale production in an economical and continuous way so far (Kadla et al. 2002; Luo et al. 2011; Mainka et al. 2015a, b; Norberg et al. 2013; Qin and Kadla 2012; Xia et al. 2016). The limited mechanical properties of both cellulose and lignin based CF, however, are not considered to be an impeding factor for certain applications such as widespread automotive use and wind energy applications which do not require the > 3GPa tensile strength offered by PAN based CF (Redelbach et al. 2012). As such, research in the development of either cellulose or lignin as precursors has reignited. Here we report on a new approach aimed at addressing both the low mass and long stabilization times of cellulose and lignin respectively by developing a composite fibre containing varying amounts of cellulose and lignin.

Using a recently developed solvent-based spinning technique, it was possible to spin continuous cellulose filaments with high molecular orientation and high mechanical properties (Hummel et al. 2015; Sixta et al. 2015). It was found, that the same solvent, 1,5-diazabicyclo[4.3.0]non-5-ene-1-ium acetate ([DBNH]OAc), is also capable of dissolving lignin of various origin (Ma et al. 2015). This opened up an entirely new approach to the utilization of lignin. Melt spinning of lignin requires a glass transition temperature below the decomposition temperature. This is often only possible through derivatization of the respective lignin (Kubo et al. 1998; Steudle et al. 2017; Uraki et al. 1995; Zhang and Ogale 2014) or by adding plasticizing agents (Kadla et al. 2002; Kubo and Kadla 2005; Saito et al. 2012). Another challenge encountered with lignin is that the polymer might differ substantially in its macromolecular properties and quality depending on the source and pulping method. Evidently, this complicates spinning of homogenous precursor filaments.

On the other hand, cellulose is available in high purity and several techniques have been established to spin continuous filaments. For further conversion into carbon fibres, non-derivatizing routes, i.e. direct dissolution of cellulose and coagulation in an anti-solvent spin bath is preferred as it produces filaments of high mechanical properties and round cross section. [DBNH]OAc is a powerful direct solvent for all wood-derived polymers. Upon co-dissolution of cellulose and lignin, the long-chain carbohydrate polymer predominately defines the visco-elastic properties of the resulting bi-polymer solution. This means, that the quality and macromolecular properties of the respective lignin is overcompensated by cellulose. The molecular weight distribution and composition of the lignin source become minor factors. Thus, a very broad set of lignin types can be used. It was possible to spin fibres with up to 50 wt.% lignin (Ma et al. 2015).

In this study, filaments with 20 and 40 wt.% organosolv lignin as model substance were investigated. Stabilization of the cellulose-lignin fibres was studied at 3 different temperatures and the fibres were characterized thoroughly in terms of thermal degradation using FTIR to determine the stabilization kinetics, evolved gases, single fibre measurements and scanning electron microscope.

## Experimental

### Materials

Birch (*Betula pendula*) prehydrolysis kraft pulp ([η] = 476 ml/g, DP = 1133, M_n_ = 65.9 kDa, M_w_ = 269.3 kDa, polydispersity 4.1, Enocell Speciality Cellulose, Finland) was delivered in sheet form and cut to a powder by means of a Willey mill. Beechwood organosolv lignin was received from Fraunhofer Institutes, Germany. 1,5-diazabicyclo[4.3.0]non-5-ene (DBN, 99%, Fluorochem, UK) and acetic acid (glacial, 100%, Merck, Germany) were used to synthesize [DBNH]OAc. The lignin-cellulose fibres where dry jet spun from a solution in [DBNH]OAc as 60-filament tow with a draw ratio of 5. Pure cellulose filaments were spun from a 13 wt.% solution. Filaments with 20 wt.% lignin (relative to cellulose) were spun from a solution with 15 wt.% polymer concentration, 40 wt.% lignin fibers from 18 wt.% polymer solution, respectively. Details regarding the synthesis of the ionic liquid, solution preparation and spinning equipment have been described previously (Ma et al. 2015). The composition of the fibres used in this study with their sample codes are summarized in Table 1.

**Table 1:** Cellulose-lignin precursor fibres used in this study

Sample ID	Composition
100C	100% cellulose
2080LC	20% Lignin, 80% Cellulose
4060LC	40% Lignin, 60% Cellulose

### Stabilization

Samples were heated in air using a Thermotec 2000 laboratory type oven at different temperatures ranging from 200 to 280 °C and times varied from 30 to 300 min. A fixed heating rate of 8 °C/min was used to raise the oven temperature from ambient temperature to the desired set temperature. The pre-treatment times were recorded after the temperature reached the set value. The effect of applying tension on stabilization was investigated by mounting the fibres on a stainless steel rig hanging with known weights varying from 17 to 34 N/m per fibre.

### Thermogravimetric analyses

Thermal stability of the lignin-cellulose fibres were measured using thermogravimetric analyses (TGA) on a TA Q50 TGA thermogravimetric analyser. Measurements were performed using 5–8 mg of the samples. The fibres were heated from 30 to 600 °C at a heating rate of 10 °C/min under N_2_ atmosphere. The thermal degradation temperature at which the weight loss begins (T_d_) was calculated as the onset. The activation energy of pyrolysis (E_k_) for fibres were calculated by the Kissinger method using the below equation (Blaine and Kissinger 2012). According to the equation, where *β*_*i*_ is the heating rate, *T*_*pi*_ is the endothermic peak temperature, *A*_*k*_ is the Arrhenius pre-exponential factor, *R* is the gas constant (8.314 J/mol K) and *E*_*k*_ is the activation energy. The sample was heated at different heating rates (*β*) of 5, 10, 15 and 20 °C/min and the endothermic peak temperature was recorded. The data plot of ln (*β/T*_*m*_^2^) versus 1/*T*_*m*_ was fitted with a linear trend line where the *E*_*k*_ was calculated from the slope of the line.

$$ \ln \left( {\frac{\beta }{{T_{m}^{2} }}} \right) = \ln \left( {\frac{{A_{k} R}}{{E_{k} }} } \right) - \frac{{E_{k} }}{R} \frac{1}{{T_{m} }} $$

### FTIR and STA-FTIR

FTIR spectra of the fibres were measured on Bruker LUMOS FTIR microscope. A thin layer of fibres was mounted on a glass slide by means of double-sided tape. The fibre layer was scanned in the frequency range of 600–4000 cm^−1^ at a scan resolution of 4 cm^−1^ with a background and sample scan time of 64 scans. FTIR measurements were repeated 6 times per sample.

The evolved gas analysis during pyrolysis of fibres was carried out using hyphenated simultaneous thermal analysis-Fourier transform infrared spectroscopy (STA-FTIR). Here, the simultaneous thermal analyser (STA 8000, Perkin Elmer) was coupled with a FT-IR spectrometer (Frontier, Perkin Elmer) via transfer line hyphenation using a TL9000 interface. Measurements were performed using approximately 3.00 mg of the samples. The specimens were heated from 30 to 600 °C at a heating rate of 10 °C/min under N_2_ atmosphere. STA data analysis was performed using the Pyris software (version 11.1.1.0492). The gasses evolved were immediately transferred to the FTIR detector through the transfer lines (balanced flow evolved gas analyser, TL9000) to continuously monitor the evolved gasses during pyrolysis. The FT-IR data were collected in the range of 4000–600 cm^−1^ at a resolution of 4 cm^−1^. Data collection and analysis was performed using Spectrum TimeBaseTM (version 3.1.3.0042) and Spectrum (version 10.4.4.449) for the time resolved IR data.

### Single fibre measurements

Tensile tests of the fibre specimens were measured using an Instron Tensile Tester fitted with a 5.00 N load cell. All tests were conducted at a fixed gauge length of 20.00 mm and at a controlled extension rate of 1.5 mm/min. The instrument was programmed to apply a pre-load of 0.10 cN before recording load-extension data. Tensile test measurements were repeated 5 times for each sample. The tensile stress of the fibres was calculated from dividing the load by the fibre cross section area, units in MPa. The cross sectional area was determine by means of an optical microscope. The Young’s modulus was obtained from measuring the gradient of the elastic region of the stress–strain curve, units in GPa. All samples were conditioned at 20 ± 2 °C and 65 ± 2% RH for 24 h prior to testing.

### Scanning electron microscope

The morphologies of the fibre cross sections were visualized with a Zeiss Supra 55VP scanning electron microscope (SEM) at an accelerating voltage of 5.00 kV. For cross section images, fibre samples were submerged in liquid nitrogen for nearly 5 min to ensure they were completely frozen. They were then removed from the liquid nitrogen bath and immediately snapped using a scalpel blade. The surface and the cleaved edge of the fibres were gold coated before observation.

## Results and discussion

### Thermal decomposition

**Fig. 1:**
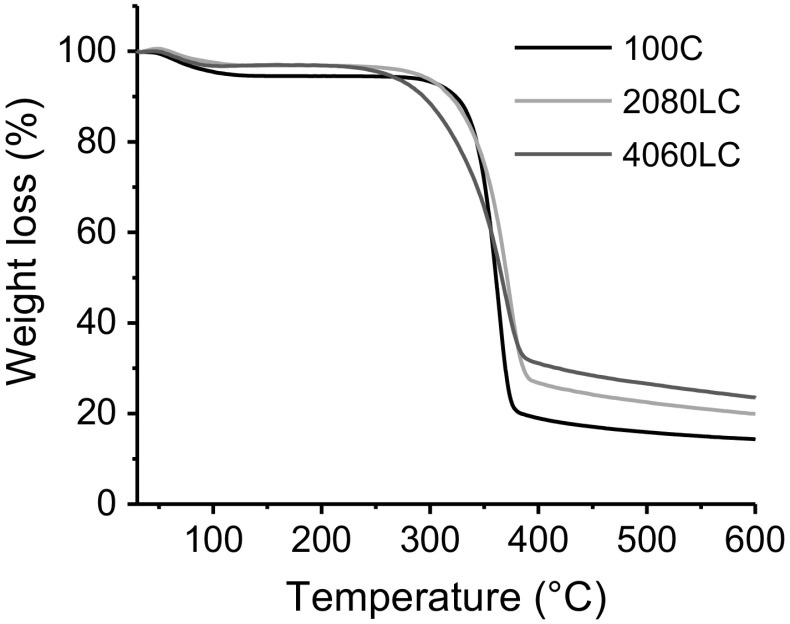
Thermogravimetric analysis curves of pure cellulose fibers and fibers with 20 and 40 wt.% lignin, respectively

Knowledge of the thermal decomposition profile of a CF precursor is critical for determining suitable stabilization parameters. Stabilization needs to be performed below the thermal decomposition temperature, T_d_. Figure 1 shows the thermal decomposition profile for the 3 precursors fibres, 100% cellulose (100C), 20% lignin: 80% cellulose (2080LC) and 40% lignin: 60% cellulose (4060LC). T_d_, mass yield at 600 °C, and activation energy for each precursor are given in Table 2. The addition of lignin lowers the T_d_ and increases the mass yield. This is expected since lignin is a high carbon content polymer and typically has a lower thermal degradation temperature as compared to cellulose (Ma et al. 2015). The addition of lignin increases the activation energy slightly.

**Table 2:** Thermal stability data obtained for cellulose lignin

Sample ID	Thermal decomposition (°C)	Mass yield (%)	Activation energy (kJ/mol)
100C	328	14.34	137.2 (0.96)
2080LC	317	19.95	141.5 (0.97)
4060LC	282	23.44	152.4 (0.96)

To shed some light on the decomposition reactions and the changes that occur upon lignin addition, the evolved gases were measured using FTIR during heating of the samples through the decomposition phase up to 600 °C. At the decomposition step, inter- and intra-molecular dehydration reactions and the cleavage of the glycosidic linkages of cellulose-lignin fibres lead to the formation of CO_2_, H_2_O and a variety of volatile carbonaceous derivatives including levoglucosan which is primarily responsible for the low mass yield of cellulose (Bacon and Tang 1964). Levoglucosan is produced via an attack of the primary hydroxyl group (–CH_2_OH) on the anomeric carbon in the cellulose polymer and particularly undesirable when aiming for a high carbon yield (Bacon and Tang 1964; Şerbănescu 2014).

**Fig. 2:**
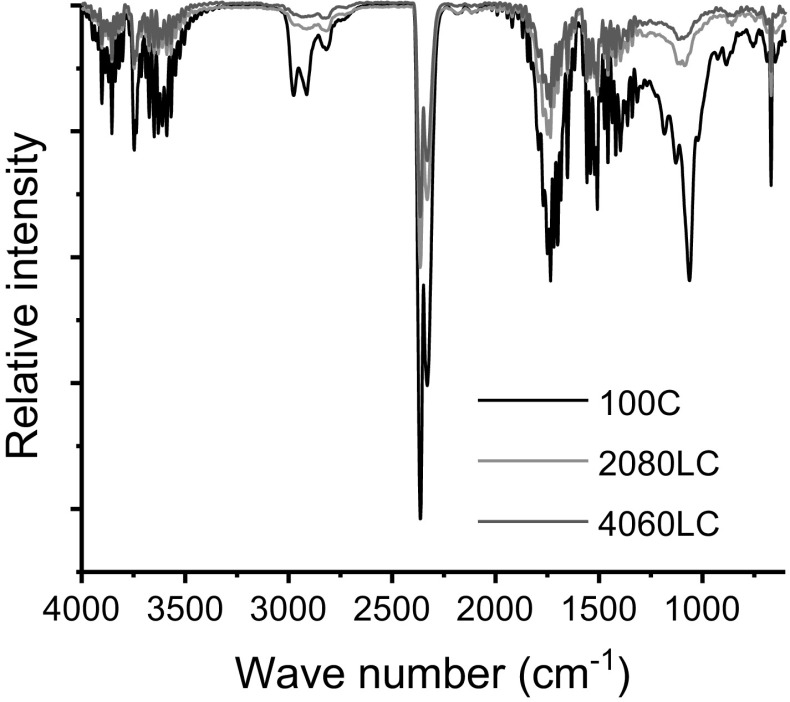
FTIR spectra of the gas phase evolved at the decomposition temperature of 100C, 2080LC, and 4060LC fibres

Figure 2 shows the IR spectra of the gaseous products formed from 100C, 2080LC and 4060LC samples, respectively, at the decomposition point of the respective fibres.

The absorption bands between 4000 and 3200 cm^−1^ were assigned to the hydroxyl groups (–OH) of the water formed during the thermal degradation. Further characteristic peaks of volatile compounds evolved during degradation were observed in the wave number region of 2400–1700 cm^−1^ including methane (CH_4_: 3017 cm^−1^), carbon dioxide (CO_2_: 2363 cm^−1^), carbon monoxide (CO: 2167 cm^−1^), and some other organic mixtures such as aldehydes (C=O: 1730 cm^−1^), and carbohydrate-typical functional groups (C–O–C/C–C: 1167 cm^−1^) (Li et al. 2001; Yang et al. 2007).

**Fig. 3:**
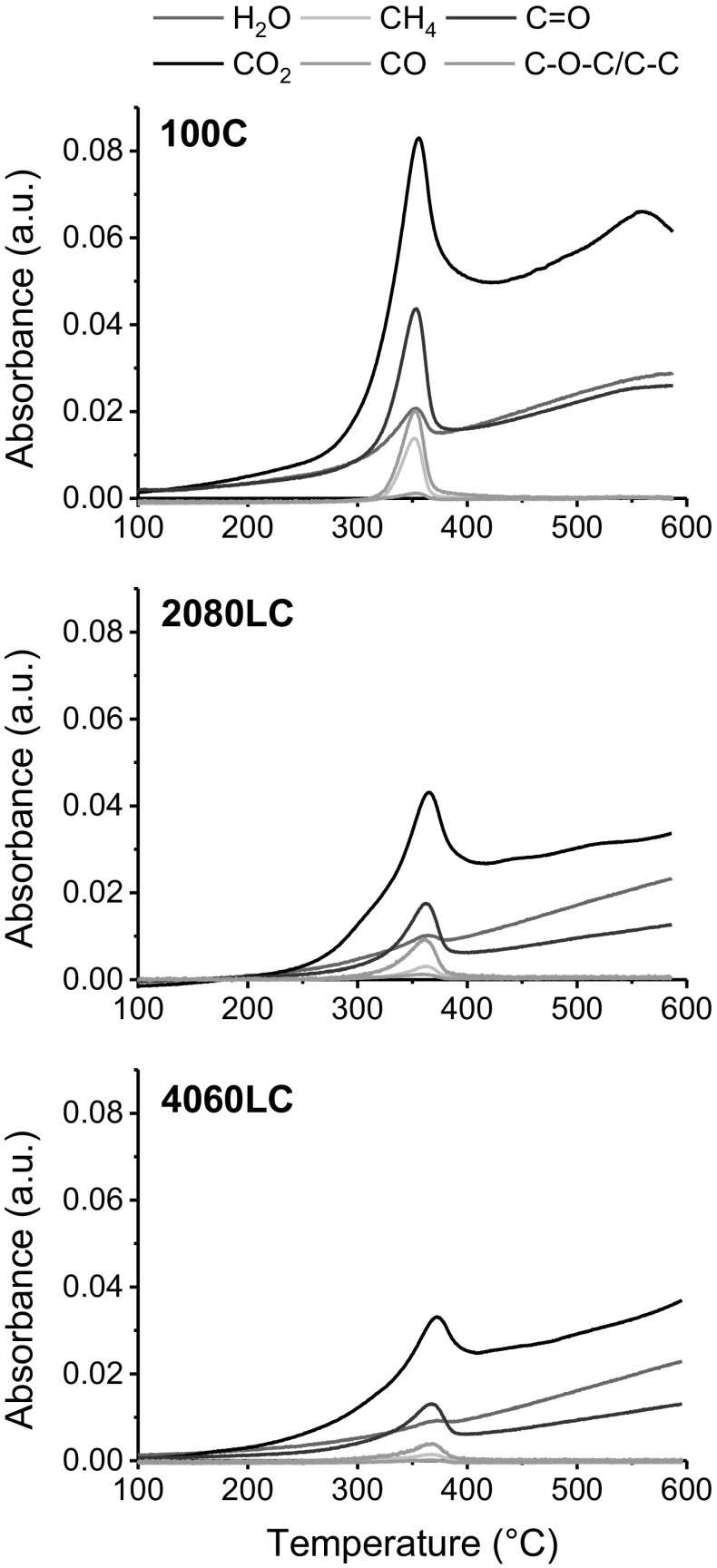
Gas evolution profiles for pure cellulose fibers (100C) and fibers with 20 (2080LC) and 40 wt.% lignin (4060LC), respectively

The STA-FTIR also allows to follow the evolution of each of the above mentioned gasses individually during the heating phase. In Fig. 3, the intensity of the IR-band assigned to a certain gas is plotted against the temperature. For all fibres, the maximum gas evolution occurred near the decomposition temperature region. Since similar amounts of the different fibre samples were weight in for TG-FTIR analysis and a uniform methodology to analyse the evolved gas was used, IR peak heights are directly proportional to the amount of released gasses (Wang et al. 2007). This allows for a semi-quantitative comparison of the different substrates regarding the evolution of volatile compounds.

Figure 3 (top) shows the main gases evolved during pyrolysis of the 100C fibre which are H_2_O, CO_2_, CH_4_, CO, aldehydes containing C=O and other carbohydrate contained functional groups of C–O–C/C–C. It has been noticed that the two major gases evolved during pyrolysis were CO_2_ and carbonylic compounds containing C=O functional groups. The evolution of CO_2_ was primarily due to the breaking and reforming of functional groups of COOH and C=O while CO is mainly evolved with the decomposition of carbonyl (C–O–C) and carbonyl (C=O) functionalities (Bacon and Tang 1964). CH_4_ evolution is likely caused by breaking methoxyl (–O–CH_3_) (Yang et al. 2007).

With the addition of lignin, more H_2_O is released relative to the total gasses produced during degradation. This formation of H_2_O during pyrolysis is highly desirable as it eliminates the primary hydroxyl group impeding the thermal cleavage of the glycosidic linkage (Brunner and Roberts 1980). This reduces the formation of levoglucosan and increases the mass yield for cellulose-lignin composite fibres as revealed by the TGA measurements. Furthermore, the total amount of released gasses was lowered when the lignin content was increased in the fibre. This suggests that the lignin component present in the composite fibre stimulates the dehydration during the pyrolysis resulting in less CO_2_, thus increasing the mass yield. This again is in good agreement with the activation energy data shown in Table 2, suggesting that such reduction in total gas evolution upon adding lignin imparts some stabilization to the fibre structure which requires more energy to decompose.

### Stabilization of cellulose-lignin composite fibres

Stabilization is a critical stage for producing carbon fibre. The purpose of the stabilization step is to create a stable fibre which can withstand the carbonization treatment. Only a successfully stabilized fibre can undergo carbonization (Rahaman et al. 2007; Ram 1970). While the precise stabilization process can be precursors specific, stabilization generally occurs at temperatures below the decomposition temperature of the precursor. During stabilization, the chemical structure of the precursor is changed through cyclization, oxidation, dehydrogenation, and cross-linking reactions. As such, structural changes and stabilization can be monitored by FTIR (Dumanlı and Windle 2012; Rahaman et al. 2007).

**Fig. 4:**
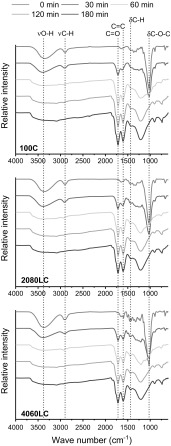
FTIR spectra of cellulose-lignin fibres stabilized at 280 °C: 100C (top), 2080LC (middle) and 4060LC (bottom)

To understand the chemical transformations occurring in the lignin/cellulose fibre during stabilization FTIR spectra were recorded at 30 min time intervals up to 180 min at a fixed temperature of 280 °C. From Fig. 4, it can be seen that all 3 precursor fibres, 100% cellulose (100C), 20% lignin: 80%cellulose (2080LC) and 40% lignin: 60% cellulose (4060LC) show characteristic cellulose bands at around 3300, 2900 and 1025 cm^−1^ attributed to O–H stretching (υO–H), C–H bending (υC–H) and pyranose ring stretching (–C–O–C) (Fan et al. 2012; Fengel and Strobel 1994). As the fibres are heated the O–H stretch band decreases likely due to a continuous dehydration process as revealed by the evolved gas analysis. As the heat treatment time increases, the appearance of two new bands at 1740 and 1620 cm^−1^ is observed. These vibrations are assigned to be C=O and C=C bonds confirming stabilization (Dumanlı and Windle 2012; Pastorova et al. 1994).

While a temperature close to the degradation temperature of the precursor allows for the reduction of the stabilization time, this occurs often at the expense of the mechanical properties. Indeed, for PAN based CFs the formation of skin–core defects is amplified at higher stabilization temperatures, attributed to an uneven heat treatment (Bahl and Manocha 1974; Kong et al. 2014; Su et al. 2013). Therefore, selecting the stabilization conditions is often a tradeoff between reducing processing times and producing quality fibre. To investigate the stabilization progress with time for the 3 precursors fibres investigated here (100C, 2080LC, 4060LC) heating experiments were conducted at 3 different temperatures 200, 240, 280 °C.

**Fig. 5:**
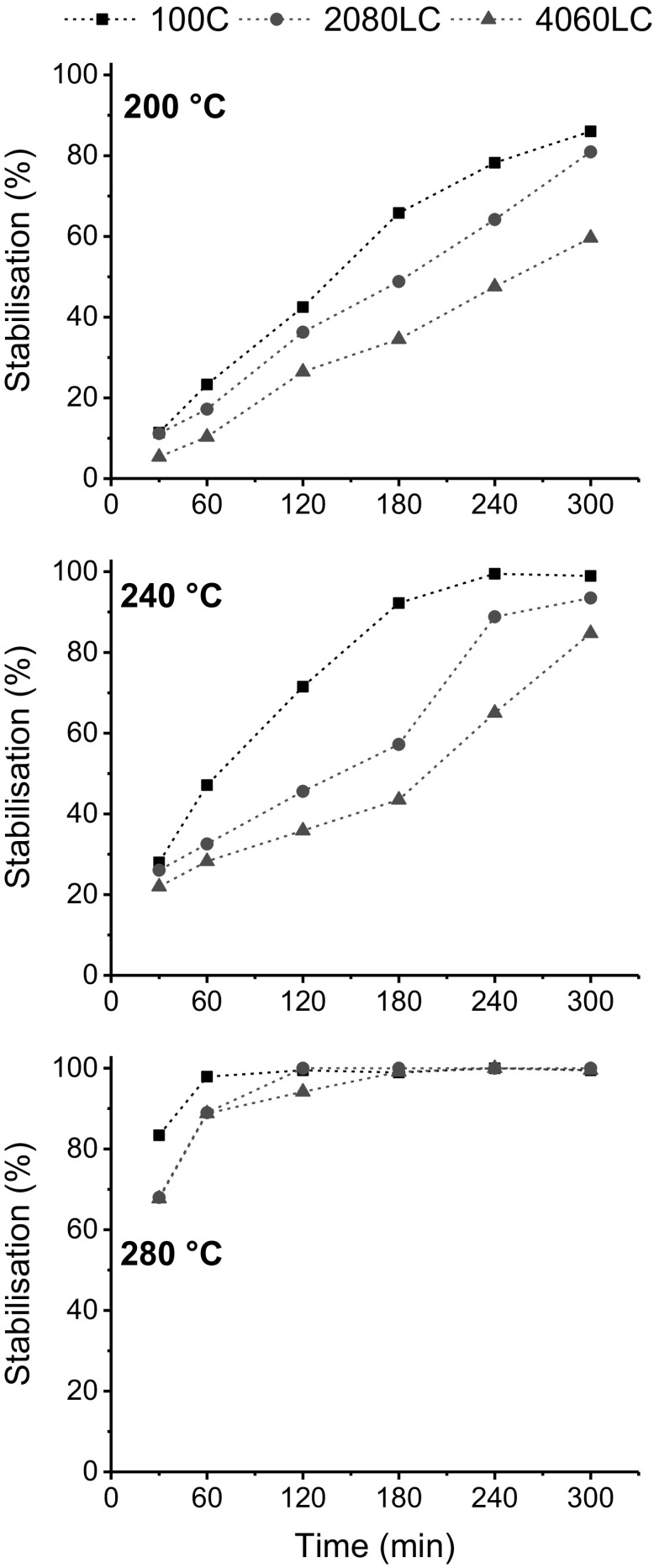
Relative stabilization of lignin/cellulose fibres treated at 200, 240, and 280 °C

The relative stabilization (expressed in %) is determined from the ratio of the FTIR-peaks and defined as I_1740cm_^−1^/I_1430cm_^−1^ (Zhang et al. 2015). Figure 5 shows the relative stabilization as a function of time at the indicated temperatures.

**Fig. 6:**
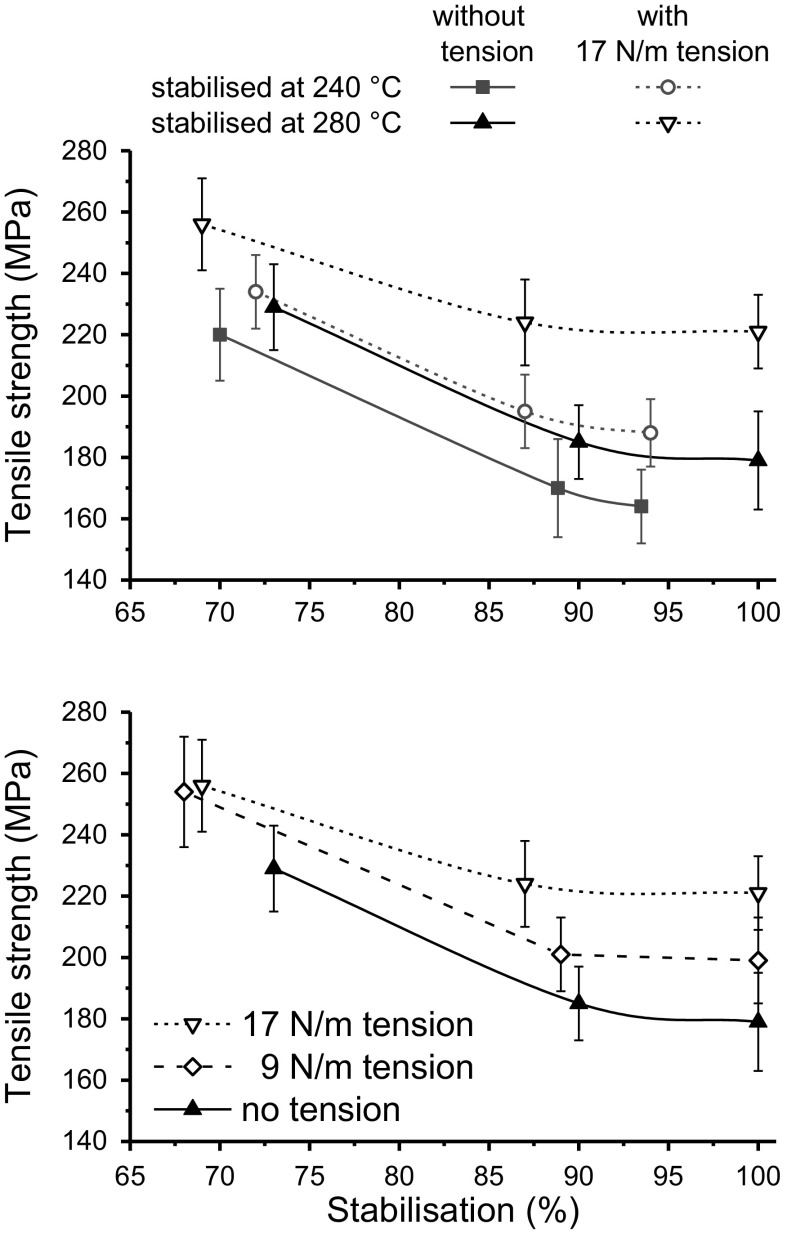
Development of the fibre tensile strength with increasing extent of stabilization. Top: 2080LC stabilized at 240 & 280 °C with and without tension, respectively; bottom: 2080LC stabilized at 280 °C with various tension

Figure 5 shows that 100C reaches stabilisation (as indicated by the plateau) after around 60 min when treated at 280 °C while 2080LC required 120 min and 4060LC required 180 min. As the stabilization temperature was reduced (Fig. 5: 200 and 240 °C, respectively) the total reaction time increased for each sample. Incomplete conversion was observed for all samples even after 300 min when heated at 200 °C. Regardless of the stabilization temperature the trend remained the same, 100% cellulose required the least intense heat treatment to reach full conversion and addition of lignin increased stabilization time gradually. Lignin is considered a very attractive renewable carbon fibre precursor. However, the stabilization of 100% lignin is currently a rate limiting step, which can take up to 100 h (Mainka et al. 2015a). As mentioned earlier the stabilization protocol is often a delicate balance between utilizing high enough temperatures and heating rates to achieve an economically sound process and perform stabilization in a sufficiently controlled manner to obtain maximal mechanical properties and reduce other potential problems such as filament fusion and skin–core effects (Brodin et al. 2012; Zhang 2016). Therefore, the impact of the stabilization temperature on the mechanical properties of the 2080LC fibres was measured at both 240 °C and 280 °C [Fig. 6 (top)]. It shows variation in tensile strength as a function of relative stabilization in the presence and absence of 17 N/m tension. Tension during the heat treatment is considered critical. For PAN based CF, tension in the furnaces enables improved alignment along the fibre axis of the graphitic domains (Guigon et al. 1984). 17 N/m was selected based on the findings shown in Fig. 6 (bottom). Here the tensile strength was measured at different relative stabilization for 2080LC using 280 °C as the stabilizing temperature applying no tension, 9 and 17 N/m. The highest tensile strength was measured for samples stabilized under 17 N/m, attempts to apply more tension resulted in breakage.

Figure 6 shows that the fibre tensile strength reduces as the stabilization progresses. This is a commonly observed trend regardless of the precursor fibre and is attributed to the changes which occur during stabilization weakening the fibre. However, it seems that a combination of a higher stabilization temperature and a tension of 17 N/m resulted in the best tensile properties for the 2080LC fibre suggesting that this precursor can withstand higher stabilization temperatures.

This would allow to reduce the stabilization time making the process economically more attractive. Table 3 shows the tensile properties obtained for 2080LC fibres stabilized at 280 °C with and without tension. The Young’s modulus also improved when applying tension whereas no clear trend was seen for the tensile strain. Generally fibre crystallinity and polymer chain orientation along the fibre axis determine the tensile strength and the Young’s modulus (Nielsen 1962; Ward 2012). This suggests that the application of tension during stabilization enhanced the fibre orientation to improve its tensile properties.

**Table 3:** Tensile properties of 2080LC fibres stabilized at 280 °C

Relative stabilization	Stabilized no tension			Stabilized with 17 N/m tension applied		
	Tensile strength (MPa)	Tensile strain (%)	Young’s modulus (GPa)	Tensile strength (MPa)	Tensile strain (%)	Young’s modulus (GPa)
68–73	229 ± 25	7.2 ± 1.1	7.6 ± 1.5	256 ± 25	7.4 ± 0.8	9.2 ± 0.6
87–90	185 ± 32	6.6 ± 1.7	7.3 ± 2.1	224 ± 23	7.1 ± 1.4	9.0 ± 1.3
100	179 ± 28	6.7 ± 2.3	6.9 ± 1.9	221 ± 23	6.4 ± 1.2	8.5 ± 1.2

**Fig. 7:**
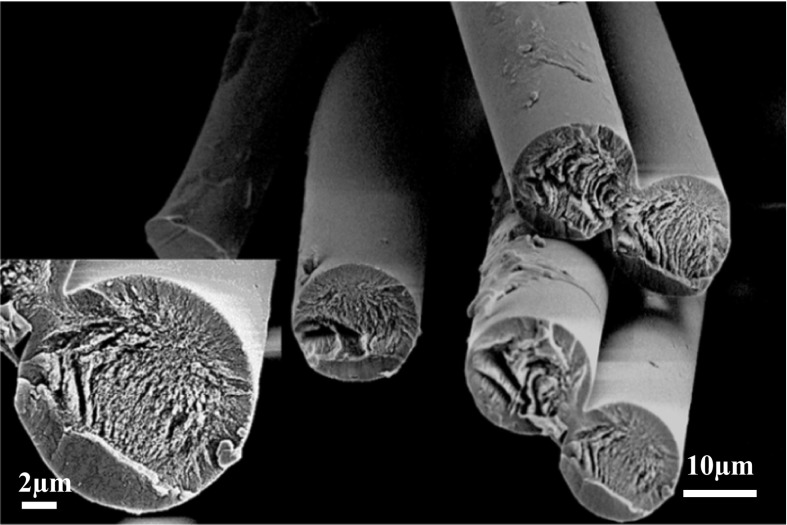
SEM image of the cross section and surface of 2080LC fibres

Figure 7 shows the cross section image of the 2080LC fibres treated at 280 °C. The stabilized fibres have a smooth surface. Some filament fusion is visible. However, this is likely already present in the precursors tow and a result of a high filament density in the dry-jet wet spinning process. Importantly, no skin–core formation is observed in the stabilized fibre. As mentioned earlier, skin–core phenomena are a crucial problem faced often with wet-spun PAN precursors. The morphological heterogeneity across the cross section deteriorates the mechanical properties of the resulting CF.

## Conclusions

This study reports a new bio based hybrid precursor fibre produced by blending cellulose with lignin and dry-jet wet spun using the ionic liquid [DBNH]OAc. The lignin-cellulose composite fibre showed a significantly higher mass yield upon pyrolysis than a pure cellulose fibre. The stabilization time of lignin is significantly decreased with the addition of cellulose. Complete stabilization of a 20:80 lignin-cellulose fibre was achieved within 2 h at 280 °C in contrast to 24 h or longer that it typically takes to stabilize pure lignin fibres. Differences in the pyrolysis mechanism with the addition of lignin were identified. In the presence of lignin a higher amount of water is released which indicates that the dehydration mechanism is favored over the undesired levoglucosan formation. By combining both cellulose and lignin in a single composite fibre limitations of the individual components in relation to carbon fibre development can be overcome. At present, the carbonization of cellulose-lignin fibres as the next phase is studied in detail.
